# *Clonorchis sinensis-*infected hepatocellular carcinoma exhibits distinct tumor microenvironment and molecular features

**DOI:** 10.3389/fimmu.2025.1526699

**Published:** 2025-03-17

**Authors:** Junxian Chen, Caibiao Wei, Wencheng Huang, Taijun Huang, Lingling Zhou, Yulong Xu, Yuling Qin, Qiumei Lin, Fengfei Liu, Xiaolan Pan, Zeli Tang, Weilong Yang, Min Fang

**Affiliations:** ^1^ Department of Clinical Laboratory, Guangxi Medical University Cancer Hospital, Nanning, China; ^2^ Department of Cell Biology and Genetics, School of Basic Medical Sciences, Guangxi Medical University, Nanning, China; ^3^ Guangzhou Women and Children’s Medical Center, Guangzhou Medical University, Guangzhou, China; ^4^ Institute of Advanced Biotechnology and School of Medicine, Southern University of Science and Technology, Shenzhen, China; ^5^ Engineering Research Center for Tissue and Organ Injury and Repair Medicine, Guangxi Medical University Cancer Hospital, Nanning, China

**Keywords:** *Clonorchis sinensis*, hepatocellular carcinoma, extracellular matrix remodeling, immunosuppression, prognosis

## Abstract

**Objectives:**

*Clonorchis sinensis* (*Cs*)-infected hepatocellular carcinoma (HCC) patients have a poorer prognosis than non-*Cs*-infected HCCs. However, the molecular mechanisms of *Cs*-infected HCC remain unclear. To address this, this study aims to uncover the tumor microenvironment and molecular features that may contribute to these poor outcomes.

**Methods:**

The research involved bulk RNA sequencing of paired tumor and adjacent tissue samples from 10 *Cs* ^+^ HCC and 10 *Cs* ^-^ HCC patients. Differentially expressed genes were identified, followed by enrichment analyses to reveal functional changes. Survival analysis of the top 10 up- and down-regulated genes in *Cs* ^+^ HCC tumors was performed using TCGA database. Additionally, clinical data from 1,461 HCC patients were retrospectively analyzed to assess the impact of *Cs* infection on microvascular invasion and metastasis rates. *In vitro* assays were also conducted using *Cs* excretory/secretory products (*Cs*ESPs) to examine their effect on HCC cells and HUVECs.

**Results:**

We identified 785 up-regulated and 675 down-regulated genes in *Cs* ^+^ HCC tumors compared to *Cs* ^-^ HCC tumors, enriched in pathways related to extracellular matrix remodeling and immunosuppression. Survival analysis revealed that the top 10 up-regulated genes are associated with HCC poor prognosis. Clinical data from 1,461 HCC patients showed *Cs* infection increased microvascular invasion and metastasis rates. *In vitro*, *Cs*ESPs products enhanced migration and invasion in HCC cells and promoted tube formation in human umbilical vein endothelial cells.

**Conclusions:**

This study provides novel insights into the molecular landscape of *Cs*-infected HCC and underscores the *Cs* infection’s role in enhancing tumor migration, invasion and angiogenesis. The findings contribute to the understanding of parasitic infections in cancer progression and suggest potential prognostic markers for *Cs* ^+^ HCC.

## 1 Introduction

Liver cancer presents as a lethal disease characterized by high prevalence and poor prognosis. It ranks as the sixth most prevalent cancer globally and the third leading cause of cancer-related fatalities (1). Hepatocellular carcinoma (HCC), the most prevalent form of liver cancer, accounts for 80% of primary liver cancer cases (2). Despite significant advancements in therapeutic interventions, most patients are diagnosed at an advanced stage, resulting in a grim prognosis for HCC (3). Therefore, it is crucial to conduct in-depth research into the complex molecular mechanisms driving HCC progression, with the aim of improving its poor prognosis.

*Clonorchis sinensis* (*Cs*), a foodborne parasitic liver fluke, is estimated to place over 200 million people worldwide at risk of infection, with more than 15 million individuals currently infected (4, 5). China bears the highest burden of *Cs* infection, contributing over 82% of global cases, with hyperendemic regions such as Guangxi Province exhibiting infection rates as high as 10.6% in recent epidemiological investigations (6, 7). *Cs* can chronically parasitize human bile ducts for extended periods, often up to 20–25 years or even lifelong (5, 8). This prolonged parasitism can accelerate the progression of hepatobiliary diseases, including cholangitis, cirrhosis, and even hepatobiliary carcinoma (4, 9, 10). During *Cs* parasitism, a wide range of compounds is secreted to elicit complex immune responses in the host, specifically through the action of excretory/secretory products produced by the parasite (*Cs*ESPs) (11). These *Cs*ESPs consist of various soluble proteins and other factors that mediate numerous interactions between humans and *Cs*, including nutrient digestion, tissue invasion, cell proliferation, and the regulation of the host’s immune system (12, 13). Recently, increasing studies have shown that *Cs* infection is significantly associated with the progression of HCC and its unfavorable prognosis (13–17). However, the specific regulatory mechanisms underlying the diminished prognosis among HCC patients with *Cs* infection have not yet been fully elucidated. Therefore, clarifying the mechanisms by which *Cs* promotes the progression of HCC and identifying potential therapeutic targets and prognostic markers, is crucial for improving the prognosis and reducing the mortality rate of patients with *Cs* -associated HCC.

With the rapid development of high-throughput technologies, RNA sequencing (RNA-seq) has become a widely used technology in the study of tumors and parasitic diseases. It is a crucial tool for uncovering gene expression patterns and molecular networks associated with these diseases. In recent years, transcriptomic analyses have been employed to study animal models of *Cs* infection. For example, in infected mouse models of *Cs*, KEGG pathway analysis of transcriptomic data has confirmed that, compared to uninfected mice, the infection is associated with pathways involved in inflammation, tumorigenesis, extracellular matrix (ECM)-receptor interactions, and metabolism (18, 19). Moreover, in a rat model of HCC with *Cs* infection, compared to HCC models alone, the significantly up-regulated differentially expressed genes (DEGs) in HCC with *Cs* infection models involve inflammation and liver fibrosis associated pathways, which acted as a potent driving force in HCC tumorigenesis and malignant progression (20). However, the clinical impact of *Cs* infection on the underlying molecular characteristics remains largely unclear. And there are currently no transcriptomic studies on human samples from HCC patients with *Cs* infection. Herein, we used RNA-seq to analyze clinical samples in order to investigate the systematic changes in gene expression induced by *Clonorchis sinensis* infection in human HCC patients, aiming to gain a deeper understanding of the effect of *Cs* infection on HCC. At the same time, we validated the RNA sequencing results, which suggested that *Cs* promotes HCC migration, invasion and angiogenesis, through retrospective analysis of clinical data and *in vitro* cellular functional experiments.

## 2 Methods

### 2.1 Human samples

Human HCC tissue samples were obtained from treatment-naive HCC patients who underwent surgical resection at the Department of Hepatobiliary Surgery, Affiliated Cancer Hospital of Guangxi Medical University (Nanning, China). All patients were informed about the procedure and signed an informed consent form. HCC tissues exhibiting characteristic macroscopic features were obtained from tumor nodules and subsequently confirmed through histological examination using hematoxylin and eosin (H&E) staining. Adjacent non-tumor tissues, free of histopathologically detectable tumor cells, were collected from regions located 5 cm away from the tumor margin.

The collection of all samples and medical data involving human subjects was approved by the Ethics Committees of the Affiliated Cancer Hospital of Guangxi Medical University (LW2024131) and conducted in accordance with the ethical principles of the Declaration of Helsinki. Upon admission, all patients gave written consent for the analysis and publication of their anonymized medical data for research purposes.

### 2.2 RNA-seq

RNA sequencing library preparation was carried out according to the manufacturer’s protocols. Total RNA was extracted using the Trizol reagent RNA extraction kit (Invitrogen, USA), and rRNA depletion was performed to enrich both mRNA and non-coding RNAs. The enriched RNA was then fragmented into short segments (~200–700 bp). First-strand cDNA synthesis was performed using a random hexamer primer, followed by second-strand synthesis using DNA polymerase I. After purification, sequencing adapters were ligated to the cDNA fragments. The second cDNA strand was selectively degraded using Uracil-N-Glycosylase (UNG). The final cDNA library was generated by Polymerase Chain Reaction (PCR), followed by DNA isolation and purification, and quantified using an Agilent 2100 (Agilent Technologies, USA). The cDNA library was sequenced on Illumina platforms in accordance with commercial protocols.

### 2.3 Analysis of RNA-seq data

Clean data was generated by removing low-quality reads and adapter sequences from raw sequencing reads using Trim Galore (v.0.6.10). The clean reads were aligned to the hg38 reference genome using Hisat2 (v. 2.2.1) with default parameters. A gene expression matrix was constructed using featureCounts (v.2.0.6). Normalization of gene expression and identification of differentially expressed genes (|Fold Change| > 2, *p* < 0.05) were performed using the R package ‘DESeq2’ (v.1.44.0). Gene Ontology (GO) and Kyoto Encyclopedia of Genes and Genomes (KEGG) enrichment analyses were conducted using the R package ‘clusterProfiler’ (v.4.12.0).

### 2.4 Protein-protein interactions analysis

The DEGs between *Cs* ^+^ HCC tumors and *Cs* ^-^ HCC tumors were used to build protein–protein interactions and STRING (21) —a protein interactome database was used in this analysis. Finally, cytoscape (v.3.7.2) was used for visualization of the protein–protein interactions networks.

### 2.5 Identify potential transcription factors

The up- and down-stream 3K sequences of up- and down-regulated genes transcriptional start sites (TSSs) were extracted, and motif enrichment analysis was performed based on the HOMER function findMotifsGenome.pl with the default option (*p* < 0.01).

### 2.6 Immunoinfiltration analysis

The R package ‘CIBERSORT’ (v.0.1.0) was utilized to assess immune infiltration by applying the principle of linear support vector regression to deconvolute the expression matrix of 22 human immune cell subtypes. Finally, the Wilcoxon test was used to identify differential immune cells.

### 2.7 Survival analysis

We selected the top 10 up-regulated and top 10 down-regulated genes between *Cs* ^+^ HCC tumors and *Cs* ^-^ HCC tumors based on fold change rankings for survival analysis using the GEPIA2 website (http://gepia2.cancer-pku.cn/#survival), utilizing the The Cancer Genome Atlas-Liver Hepatocellular Carcinoma (TCGA-LIHC) cohort with default parameters. A *p*-value of less than 0.05 was considered significant, indicating an association between the mRNA and the survival prognosis of HCC patients.

### 2.8 Study population and data collection

We meticulously gathered data from 1,461 HCC patients who were diagnosed at the Tumor Hospital of Guangxi Medical University between July 2013 and December 2022. All these patients underwent curative resection. The criteria for inclusion in this retrospective study were (1): HCC confirmed through postoperative pathological analysis, (2) patients consented to post-operative follow-up visits, (3) all patients underwent testing for *Cs* infection at the time of initial diagnosis. The exclusion criteria were: (1) patients who had received previous anti-tumor treatments such as radiotherapy or chemotherapy, (2) cases lacking a definitive pathological diagnosis, (3) patients with other tumor-related disease, (4) those with recurrent HCC, and (5) incomplete laboratory, follow-up data and died perioperatively.

The diagnostic criteria for infection with *Cs* are as follows (14, 22): (1) Preoperative imaging (MRI, CT, microscopy, or ultrasound) showing *Cs* eggs or adult worms in the intrahepatic bile ducts; (2) Intraoperative or postoperative pathological examination detecting adult *Cs* in the liver or gallbladder; (3) Preoperative fecal examination revealing *Cs* eggs.

Data collection was performed by two independent investigators, JXC and CBW, with validation by a third investigator, TJH. The data collection included the pathological characteristics of liver resection specimens from 1,461 HCC patients, focusing on indicators such as the presence of MVI and Ki67 positivity rate. Clinical characteristics of patients in the retrospective study can be found in **Supplementary Table S1**.

### 2.9 Histologic and immunohistochemistry information

All pathological specimens of postoperative liver cancer were fixed in formalin and subsequently embedded in paraffin wax, then sectioned into continuous slices of 4 μm thickness. Hematoxylin and eosin (HE) staining revealed clusters of tumor cells within the lumen of endothelial-lined blood vessels, primarily located in the peritumoral portal vein branches (including vessels within the capsule), confirming the presence of MVI. To further investigate cellular proliferation, immunohistochemical analysis was performed using Ki67 mouse anti-human monoclonal antibody (dilution 1:200; Maixin Biotech, China). After deparaffinization, the sections underwent high-pressure antigen retrieval in citrate buffer (pH 6.0), followed by treatment with 3% H_2_O_2_ to eliminate endogenous peroxidase activity. Normal non-immune sheep serum was then used to block the sections, reducing nonspecific binding. The sections were subsequently incubated overnight at 4℃ with the primary antibody. Finally, the DAKO EnVision detection system was used for visualization, with phosphate-buffered saline (PBS) serving as the negative control to ensure the reliability of the experimental results.

According to the microscopic observation of microvascular invasion (MVI) results, the presence of MVI is identified when there are more than 5 instances of MVI in postoperative liver cancer tissue, or when MVI occurs in the distant cancer-adjacent liver tissue region (> 1 cm).

Ki67 is primarily expressed in the cell nucleus, with positive results appearing as yellow or brownish-yellow staining. The scoring method for Ki67 is based on the Ki67 index, which refers to the percentage of tumor cells with stained nuclei relative to the total number of tumor cells observed.

All diagnoses of Ki67 and MVI were independently confirmed by two pathologists. In cases of discrepancy, slides were reviewed by a pathologist with a title of associate chief physician or higher, and the diagnosis of Ki67 and MVI was jointly confirmed by all three pathologists.

### 2.10 Follow-up routine and calculation of metastasis rates

All patient follow-up data are carefully managed by healthcare professionals. Disease status and the date of metastasis are determined through telephone follow-up or outpatient monitoring systems. Tumor recurrence and metastasis are assessed based on radiological findings from CT or MRI scans, combined with pathological examination results. Using this information, the metastasis rates of *Cs*-infected and non-*Cs*-infected HCC patients were compared and analyzed.

### 2.11 Collection and preparation of *Cs*-produced excretory/secretory products

*Cs* adult worms were washed several times with PBS (Gibco, USA) containing 1% Penicillin-Streptomycin solution (Solarbio, China) and then cultured in glass dishes using a phenol red-free 1640 medium (Solarbio, China). After 48 hours, the culture medium was collected and centrifuged at 4,000 rpm for 30 minutes at 4°C to remove eggs and cell debris. The resulting supernatant was then centrifuged at 12,000 rpm for 45 minutes at 4°C to obtain the supernatant. The supernatant was dialyzed against PBS to remove small molecules and then concentrated using sucrose or freeze-dried for further analysis. The molecular weight and concentration of the ESPs were measured, and the samples were stored at -80℃. Before use, the samples were sterilized via 0.22 μm filtration to ensure they were free from contaminants.

### 2.12 Cell culture

Human umbilical vascular endothelial cells (HUVECs) and human HCC cell lines MHCC-97H were obtained from the Type Culture Collection of the Chinese Academy of Sciences (Shanghai, China). MHCC-97H cells were cultured in high-glucose Dulbecco’s Modified Eagle Medium (DMEM) (Gibco, USA) supplemented with 10% fetal bovine serum (FBS) (Wisent, Canada) and 1% Penicillin-Streptomycin solution (Solarbio, China). HUVECs were cultured in endothelial cell medium (ECM) supplemented with endothelial cell growth supplement (ECGS) and 5% fetal bovine serum (FBS) (ScienCell Research Laboratories, USA), and utilized for experiments between passages 4 and 8. In the experimental group, the concentration of *Cs*ESPs was set at 50 μg/ml, while the control group received an equivalent volume of 1×PBS (Gibco, USA) solution. Cells were incubated at 37℃ in a humidified atmosphere containing 5% CO_2_ and 95% air.

### 2.13 Cell proliferation

Cell proliferation was evaluated using the Cell Counting Kit-8 (CCK8) assay. Briefly, cells were seeded at a density of 2×10³ cells/100 μL per well in 96-well plates. In the experimental group, the concentration of *Cs*ESPs was set at 50 μg/mL, while the control group received an equivalent volume of 1×PBS. On Days 0, 1, 2, and3, the medium was replaced with 100 μL of serum-free medium in each well, followed by the addition of 10 μL of CCK8 reagent (UElandy, China). The plates were then incubated for 1 hour, and absorbance was measured at 450 nm using a microplate reader to assess relative cell proliferation.

### 2.14 Wound healing assay

Six-well plates were used to seed MHCC-97H cells, which were then incubated for 24 hours in DMEM containing 1% FBS. All cells were maintained at 37°C in a humidified incubator with 5% CO_2_. Each well was inoculated with 5×10^5^ cells, ensuring thorough mixing during the seeding process. When the cells reached 80% to 90% confluence, the culture medium was removed, and the floating cells were carefully washed twice with 1×PBS (Gibco, USA). Using a 10 μL pipette tip, three horizontal scratches were made across each well. The wells were subsequently rinsed with 1×PBS to eliminate any remaining floating cells. Once the background was clean, the cells were cultured in medium containing 1% FBS and treated with different substances, with 2 ml of medium added to each well. In the experimental group, the concentration of *Cs*ESPs was set to 50 μg/ml, while the control group received an equal volume of 1×PBS solution. Imaging was performed using a light microscope (ZEISS Axio Vert.A1, Germany) immediately after adding the drug-containing medium (0 hours), followed by additional observations and imaging at 24-hour intervals until 72 hours.

### 2.15 Transwell assay

The migration and invasion capabilities of MHCC-97H cells through filters were assessed using Transwell chambers (Costar, USA). Briefly, MHCC-97H cells co-cultured with *Cs*ESPs or PBS were serum-starved for 24 hours. Afterward, 1×10^6^ cells/well in 200 μL of serum-free DMEM were placed in the upper chamber at 37°C. For the matrix invasion assay, Matrigel (Corning, USA) was added to the upper chamber in a ratio of 1:8 with serum before the cells were introduced., while for the migration assay, Matrigel was not included. 600 μL of DMEM containing 20% FBS was added to the lower chamber. After 48 hours of incubation, the cells that migrated to the bottom surface of the membrane were washed with PBS, fixed with formaldehyde for 30 minutes, stained with 0.5% crystal violet (Solarbio, China), and counted. The migration and invasion cells located at the bottom of each chamber were photographed and quantified using a microscope (ZEISS Axio Vert.A1, Germany). Cell counts were randomly performed in three high-power fields.

### 2.16 Tube formation assay in Matrigel

*In vitro* capillary network formation was determined by performing a tube formation assay in Matrigel (Corning, USA). Human umbilical vein endothelial cells (HUVECs) co-cultured with *Cs*ESPs or PBS were plated in triplicate on 50 uL Matrigel -coated 96-well plate, with 1.5 × 10^4^ cells per well in 100 μL of medium. After 8h of incubation, tube formation was examined by microscopy (ZEISS Axio Vert.A1, Germany), and the tube numbers and tube length were quantified by randomly selecting three fields per well using the lmageJ program. Higher values of these parameters indicate greater quantity and quality of tube formation.

### 2.17 Statistical analysis

GraphPad Prism 9.5.0 software was used for statistical analysis. Intergroup differences in categorical data presented as ratios were compared using the Chi-square test. Continuous variables were analyzed using the Student’s *t*-test and ANOVA. All experimental results are based on three independent experiments, presented as means ± standard deviation (SD), with *p* < 0.05 considered statistically significant.

## 3 Results

### 3.1 The RNA profiles in *Cs^+^* HCC

**Figure 1:**
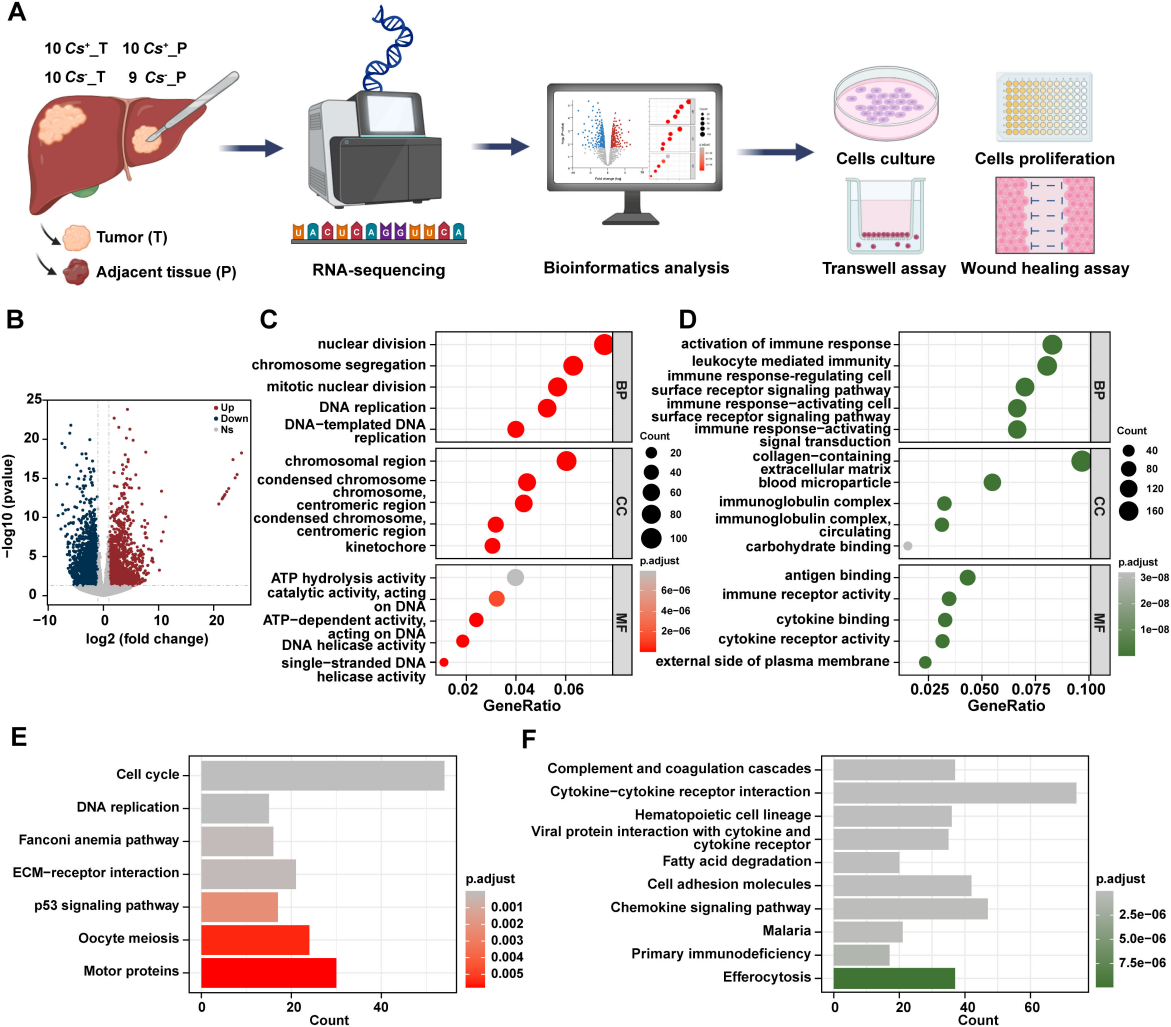
Distinct RNA profiles between *Cs^+^* HCC tumors and adjacent non-tumor tissues. **(A)** Schematic overview of the analytical workflow and experimental design of this study. Created with BioRender.com. **(B)** Volcano diagram of DEGs between *Cs^+^* HCC tumors and adjacent non-tumor tissues. GO terms for up **(C)** and down **(D)** -regulated genes between *Cs^+^* HCC tumors and adjacent non-tumor tissues. KEGG analysis of up **(E)** and down **(F)** -regulated genes between *Cs^+^* HCC tumors and adjacent non-tumor tissues.

To explore the tumor microenvironment and molecular features of *Cs*-infected HCC, an analytical workflow and experimental design was created (**Figure 1A**). Bulk RNA sequencing (RNA-seq) was conducted on samples collected from 10 *Cs*-positive HCC patients, comprising 10 cases of tumor tissue (*Cs^+^* _T) and 9 cases of adjacent non-tumor tissue (*Cs^+^* _P), with one adjacent non-tumor sample excluded due to unsatisfactory quality. Additionally, RNA-seq was conducted on samples collected from 10 *Cs*-negative HCC patients, including 10 pairs of tumor tissue (*Cs* ^-^_T) and their corresponding adjacent non-tumor tissue (*Cs* ^-^_P). All of which were obtained from treatment-naive HCC patients through surgical resection. Details for all patients are provided in **Supplementary Table S2**. The gene expression levels of all samples are shown in **Supplementary Table S3**.

To start with, we investigated the RNA expression levels of *Cs^+^* HCC and identified the differentially expressed genes (DEGs) between tumors and their adjacent non-tumor tissues. Totally, 1,984 up-regulated genes and 2,010 down-regulated genes were found in *Cs^+^* HCC tumors compared to adjacent non-tumor tissues (**Figure 1B**). Gene Ontology (GO) analysis revealed that up-regulated genes were enriched in biological processes such as nuclear division, cellular components like chromosomal region, and molecular functions such as ATP hydrolysis activity, etc (**Figure 1C**), and down-regulated genes were enriched in biological processes related to activation of immune response, cellular components such as collagen-containing extracellular matrix, and molecular functions like antigen binding, etc (**Figure 1D**). Additionally, the KEGG analysis revealed that up-regulated genes were significantly enriched in pathways such as cell cycle, DNA replication, and ECM-receptor interaction, etc (**Figure 1E**), and down-regulated genes were enriched in pathways including complement and coagulation cascades, chemokine signaling pathway, and cell adhesion molecules, etc (**Figure 1F**).

### 3.2 The RNA profiles in *Cs^-^* HCC

**Figure 2:**
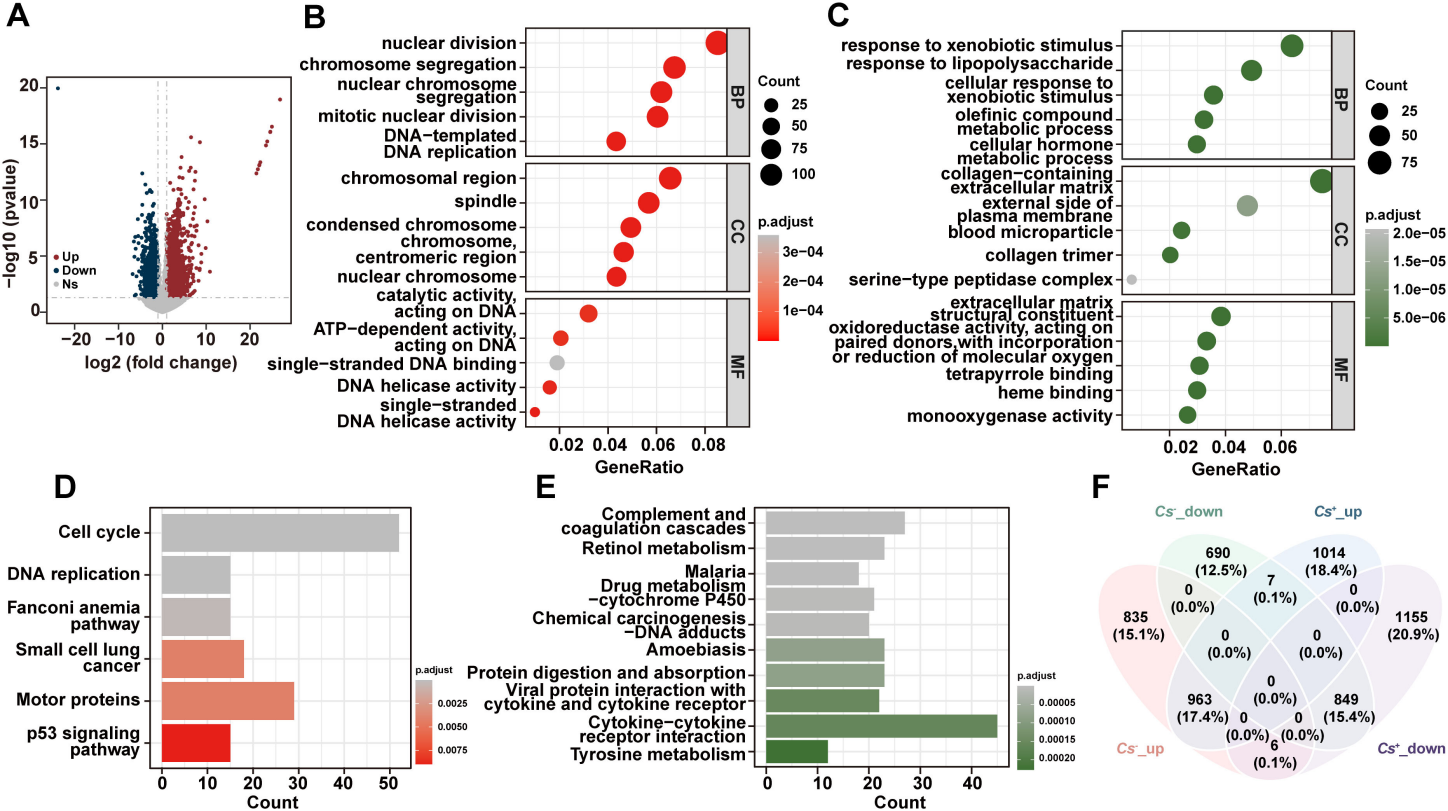
Distinct RNA profiles between *Cs* ^-^ HCC tumors and adjacent non-tumor tissues. **(A)** Volcano diagram of DEGs between *Cs* ^-^ HCC tumors and adjacent non-tumor tissues. Enrichment analysis of GO terms for up **(B)** and down **(C)** -regulated genes between *Cs* ^-^ HCC tumors and adjacent non-tumor tissues. KEGG analysis of up **(D)** and down **(E)** -regulated genes between *Cs* ^-^ HCC tumors and adjacent non-tumor tissues. **(F)** Venn diagram for the relationship between DEGs in *Cs^+^* and *Cs* ^-^ HCC.

In addition, the DEGs between tumors and adjacent non-tumor tissues in *Cs^-^* HCC were analyzed, and 1,804 up-regulated genes and 1,546 down-regulated genes were found in *Cs^-^* HCC tumors compared to adjacent non-tumor tissues (**Figure 2A**). The GO analysis reveals that up-regulated genes are mainly enriched in nuclear division, chromosomal regions, and catalytic activity, acting on DNA, etc (**Figure 2B**), and down-regulated genes are primarily enriched in response to xenobiotic stimuli, collagen-containing extracellular matrix, and extracellular matrix structural constituents, etc (**Figure 2C**). The KEGG analysis revealed that up-regulated genes were significantly enriched in cell cycle, DNA replication, p53 signaling pathway, and motor proteins, etc (**Figure 2D**), and down-regulated genes were mainly enriched in cytokine-cytokine receptor interaction, complement and coagulation cascades and retinol metabolism, etc (**Figure 2E**). Finally, we also compared the up-regulated and down-regulated genes between tumors and adjacent tissues in *Cs^+^* and *Cs^-^* HCC. The results revealed a significant difference, suggesting that *Cs* infection remodels the gene expression landscape in HCC (**Figure 2F**).

### 3.3 *Cs* infection induces different RNA expression profiles of tumors in HCC

**Figure 3:**
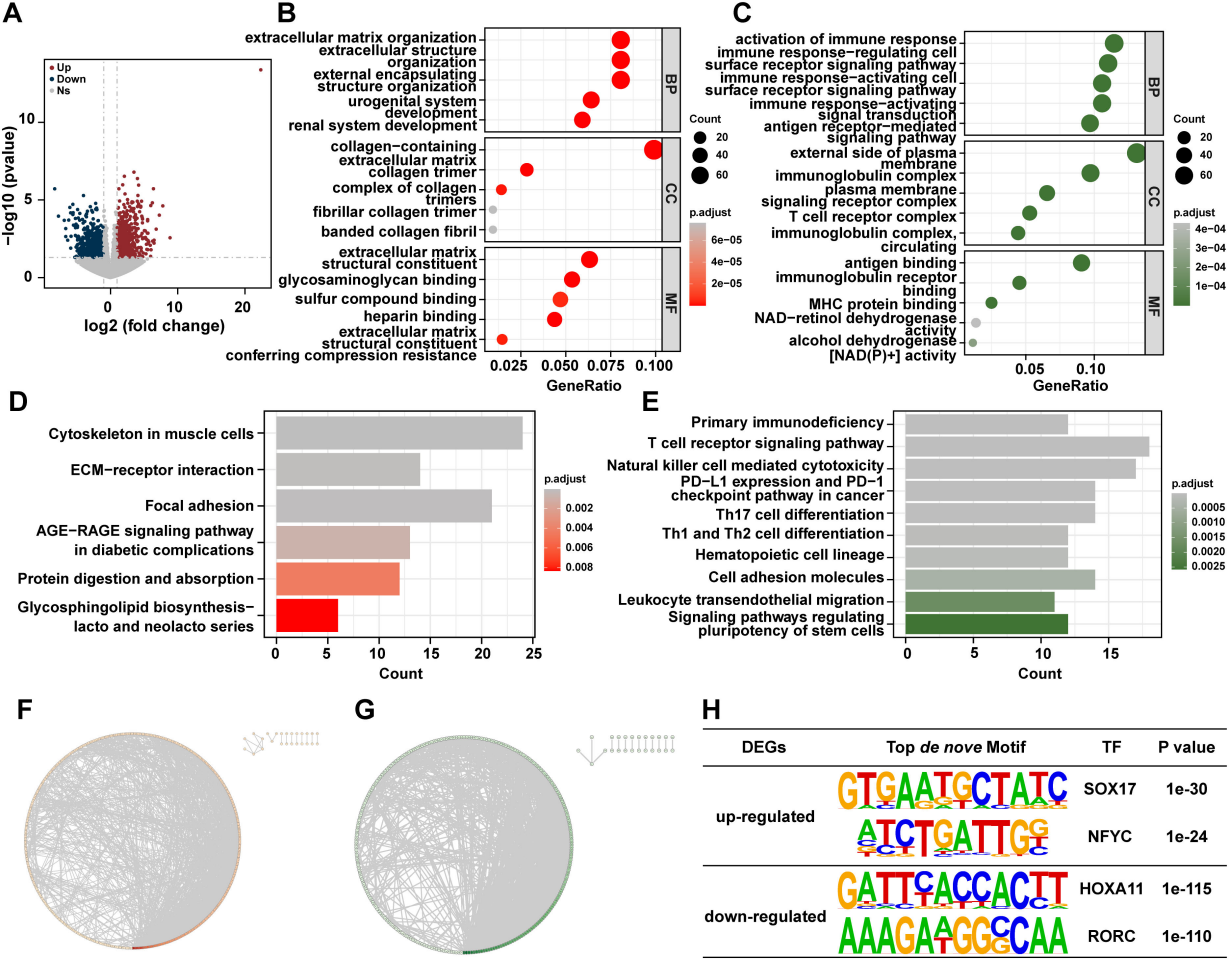
Distinct RNA profiles between *Cs* ^+^ HCC tumors and *Cs* ^-^ HCC tumors. **(A)** The volcano plot shows DEGs of *Cs* ^+^ HCC tumors and *Cs* ^-^ HCC tumors. Enrichment analysis of GO terms for up **(B)** and down **(C)** -regulated genes between *Cs* ^+^ HCC tumors and *Cs* ^-^ HCC tumors. KEGG analysis of up **(D)** and down **(E)** -regulated genes between *Cs* ^+^ HCC tumors and *Cs* ^-^ HCC tumors. Protein-protein interaction (PPI) network of up **(F)** and down **(G)** regulated genes between *Cs* ^+^ HCC tumors and *Cs* ^-^ HCC tumors. **(H)** The top *de novo* motifs enriched in up and down -regulated genes between *Cs* ^+^ HCC tumors and *Cs* ^-^ HCC tumors.

To further explore the molecular differences between malignant cells in the two groups, the mRNA expression profiles of *Cs^+^* HCC tumors and *Cs^-^* HCC tumors were compared. 785 up-regulated genes and 675 down-regulated genes were found in *Cs^+^* HCC tumors compared to *Cs^-^* HCC tumors (**Figure 3A**). The GO analysis reveals that up-regulated genes are mainly enriched in extracellular matrix organization, collagen-containing extracellular matrix and extracellular matrix structural constituent, etc (**Figure 3B**), and down-regulated genes are primarily enriched in response to activation of immune response, external side of plasma membrane, and antigen binding, etc (**Figure 3C**). The KEGG analysis revealed that up-regulated genes were significantly enriched in Cytoskeleton in muscle cells, Focal Adhesion, ECM-receptor interaction, Focal adhesion, etc (**Figure 3D**), and down-regulated genes were mainly enriched in T cell receptor signaling pathway, natural killer cell mediated cytotoxicity, PD-L1 expression and PD-1 checkpoint pathway in cancer, etc (**Figure 3E**). This suggests that *Cs* infection may promote HCC progression involved ECM remodeling and immune responses pathway. Furthermore, to investigate the interactions among the DEGs, we constructed a PPI network (**Figures 3F, G**). 2034 and 2063 gene interactions were found in up- and down-regulated genes, respectively. Moreover, top 3 interaction genes were COL1A1, COL1A2 and COL3A1 in up-regulated genes, and top 3 interaction genes were CD2, CD4 and CD19 in down-regulated genes. Last but not least, motif analysis was performed on the regulatory regions of up-regulated and down-regulated genes. SOX17 and NFYC were enriched in the regulatory regions of up-regulated genes and HOXA11 and RORC were enriched in the regulatory regions of down-regulated genes, respectively (**Figure 3H**).

Considering the GO and KEGG results found that the DEGs between *Cs^+^* HCC tumors and *Cs^-^* HCC tumors were involved in tumor immune regulation, we next assessed the immune microenvironment in the *Cs^+^* HCC tumors and *Cs^-^* HCC tumors. The immune infiltration analysis showed that resting dendritic cells (DCs) were significantly more abundant in *Cs^+^* HCC tumors compared to *Cs^-^* HCC tumors (*p* < 0.05) (**Supplementary Figure S1A**). Additionally, the T-cell stimulant-related genes CD27, ICOS, and TNFRSF8 were significantly down-regulated in *Cs^+^* HCC tumors (**Supplementary Figure S1B**). However, there were no significant differences in the HLA-related genes between *Cs^+^* HCC tumors and *Cs^-^* HCC tumors (**Supplementary Figure S1C**).

Increasing evidence suggests that the progression of HCC is closely linked to metabolic reprogramming (23, 24). Metabolic pathway events encompass the Warburg effect and an increased rate of oxidative phosphorylation (OXPHOS), which supply energy for the growth and invasion of cancer cells (25–27). These metabolic alterations not only support the rapid proliferation and survival of tumor cells but may also influence the aggressiveness of the tumor and its resistance to treatment (28). Therefore, we also compared the differential genes in this metabolic pathway between *Cs^+^* tumors and *Cs^-^* tumors. We found that genes associated with OXPHOS, such as complex I (MT-ND1, MT-ND2 NDUFA4L2 and NDUFS3), IV (COX7A2L), and V (ATP6V1E1 and ATP6V1H) of the electron transport chain were up-regulated in *Cs^+^* tumors, while gene related to complex I (NDUFA8) was down-regulated in *Cs^+^* tumors (**Supplementary Figure S2**).

Given that lncRNAs play crucial regulatory roles in various biological events related to tumorigenesis and development, we also compared the differential gene expression of lncRNAs between *Cs^+^* HCC tumors and *Cs^-^* HCC tumors. 303 up-regulated genes and 286 down-regulated genes of lncRNA level were found in *Cs^+^* HCC tumors compared to *Cs^-^* HCC tumors (**Supplementary Figure S3**).

To explore whether *Cs* infection also changed RNA expression profiles of adjacent non-tumor tissues, the relevant analyses were performed. 696 up-regulated and 663 down-regulated genes were found in *Cs^+^* HCC adjacent non-tumor tissues compared to *Cs^-^* HCC adjacent non-tumor tissues (**Supplementary Figure S4A**). The GO analysis reveals that up-regulated genes are mainly enriched in leukocyte migration, collagen-containing extracellular matrix, AGE-RAGE signaling pathway and receptor ligand activity, etc (**Supplementary Figure S4B**). Moreover, the KEGG analysis revealed that up-regulated genes were significantly enriched in Cytokine-cytokine receptor interaction, PI3K-Akt signaling pathway, and focal adhesion, etc (**Supplementary Figure S4C**).

### 3.4 The DEGs between *Cs* ^+^ HCC tumors and *Cs* ^-^ HCC tumors have clinical relevance in LIHC-TCGA cohort

**Figure 4:**
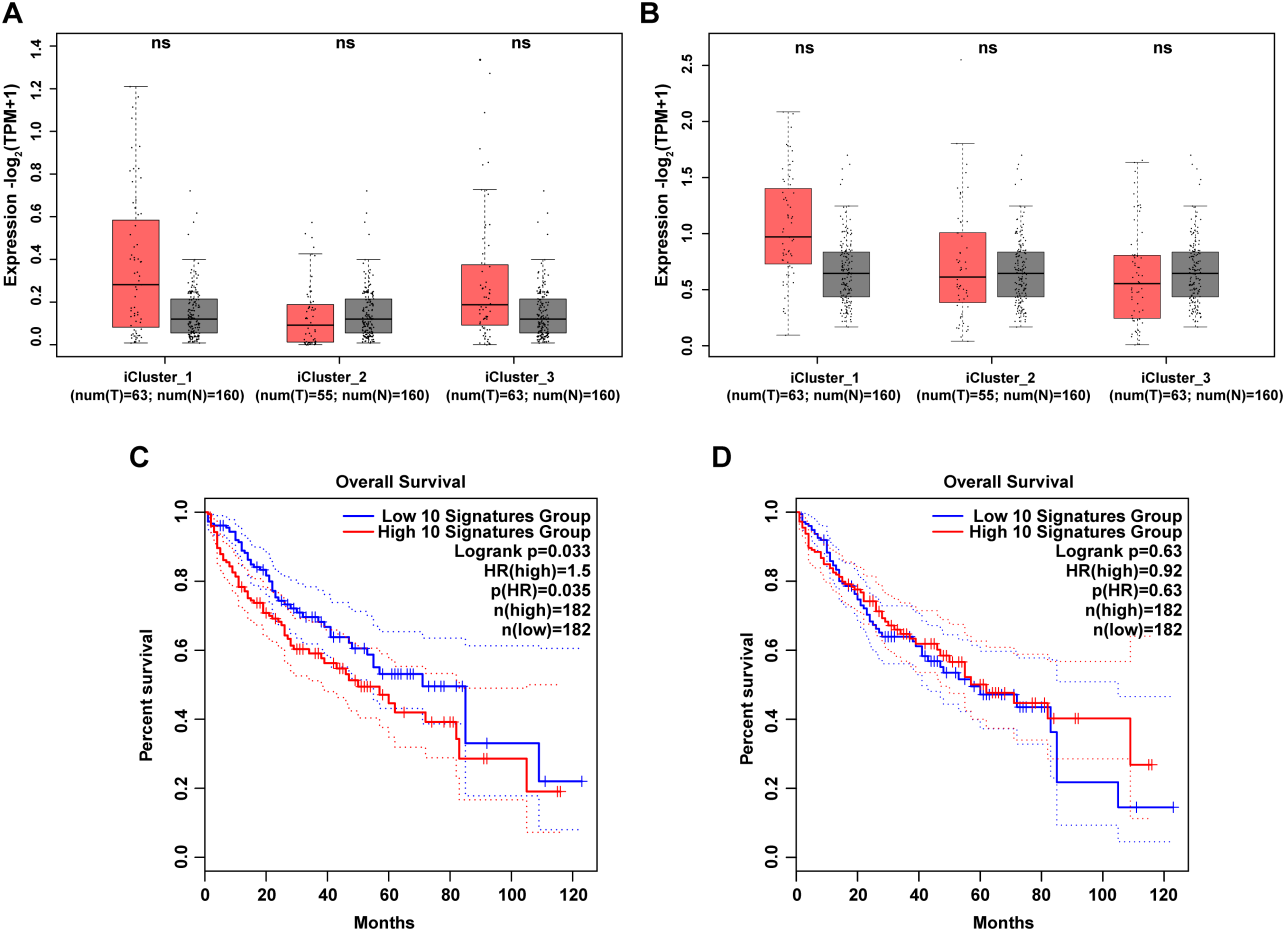
Clinical relevance of DEGs between *Cs^+^* HCC tumors and *Cs^-^* HCC tumors in LIHC-TCGA cohort. **(A, B)** Box plots illustrate the grouped expression levels of the top 10 up **(A)** and down **(B)** -regulated genes between *Cs^+^* tumors and *Cs^-^* tumors in three subtypes of LIHC tumors and their corresponding adjacent normal tissues. **(C, D)** Kaplan-Meier plots present the OS curves of LIHC patients classified into high and low signature scores for the top 10 up **(C)** and down **(D)** -regulated genes as described above.

Next, we screened the top 10 up-regulated and down-regulated genes in *Cs* ^+^ HCC tumor tissues compared to *Cs* ^-^ HCC tumor tissues based on our clinically collected cohort and further investigated the expression characteristics of these genes in the TCGA-LIHC dataset. The top 10 up-regulated genes are CLVS2, IBSP, GP2, CXCL5, LINC00348, SMYD1, BPIFB1, CLDN18, SULT1C3 and GABRG3. And the top 10 down-regulated genes are MYH4, GATA5, ODAM, POU6F2, ZIM2, GFRA3, IGKV3-11, IGLC7, COL2A1 and NPFFR1. As shown in **Figures 4A, B**, the grouped expression levels of the top 10 up-regulated and down-regulated genes show no significant differences across the three subtypes of LIHC. However, when analyzing the association between the signature scores of the top 10 up-regulated and down-regulated genes and overall survival (OS) in LIHC patients, we found a correlation between these scores and specific clinical outcomes in LIHC. High expression level of grouped top 10 up-regulated genes are associated with shorter OS in LIHC-TCGA cohort (*p* < 0.05) (**Figure 4C**). And low expression levels of the grouped top 10 down-regulated genes to be associated with shorter OS, although the differences are not statistically significant (**Figure 4D**). Consequently, our survival analysis results suggests that the DEGs between *Cs^+^* HCC tumors and *Cs^-^* HCC tumors have clinical relevance in LIHC-TCGA cohort.

### 3.5 *Cs* infection could promote HCC migration, invasion and angiogenesis

**Figure 5:**
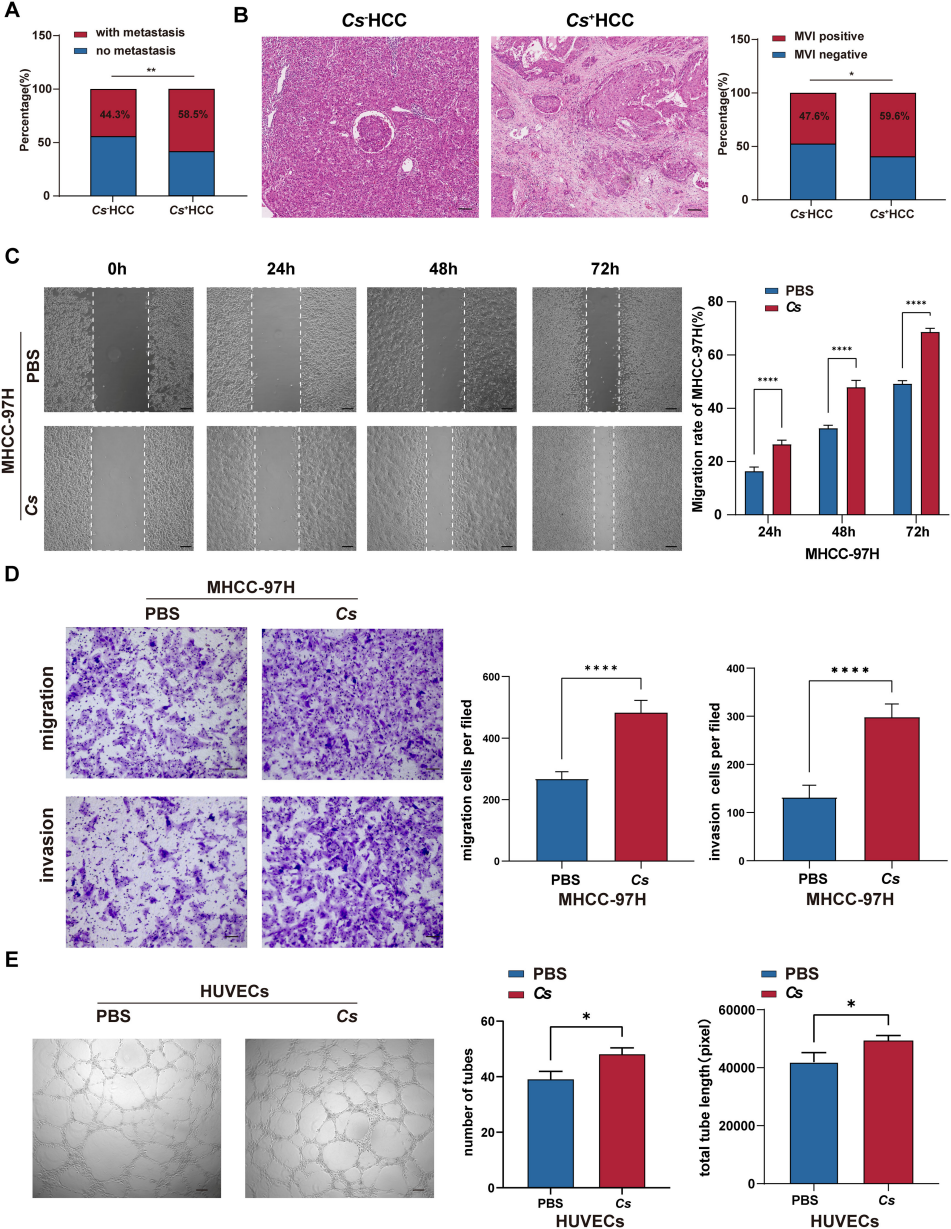
Verify the effects of *Cs* infection on invasion and migration in HCC. **(A)** Comparison of metastasis rate between *Cs* ^+^ (*n *= 94) and *Cs^-^* (*n *= 1,367) HCC patients. **(B)** Comparison of MVI positivity rate between *Cs* ^+^ (*n *= 94) and *Cs^-^* (*n *= 1,367) HCC patients, representative HE staining image showing the presence of MVI (Scale bar: 50 μm). **(C)** Transwell assay of MHCC-97H cells co-cultured with *Cs*ESPs or PBS (Scale bar: 200 μm) (*n *= 3). **(D)** The scratch assay of MHCC-97H cells co-cultured with *Cs*ESPs or PBS (Scale bar: 50 μm) (*n *= 3). **(E)** Tube formation assay of HUVECs co-cultured with *Cs*ESPs or PBS (Scale bar: 200 μm) (*n *= 3). **(C-E)** Data are represented as mean ± SD, *p < 0.05, **p < 0.01, ****p < 0.0001.

The above analysis found that *Cs^+^* HCC tumors are related to ECM-receptor interaction and focal adhesion (**Figure 3**). It is well-known that ECM derived from tumor cells is crucial in cancer progression, as its remodeling through synthesis and degradation facilitates cell adhesion and the infiltration of endothelial and tumor cells, promoting angiogenesis and leading to metastasis (29). This suggests that *Cs* infection may promote HCC migration, invasion and angiogenesis. In order to confirm the above hypothesis, we conducted a retrospective analysis of 1,461 treatment-naive HCC patients who underwent surgical resection from January 2013 to December 2022, focusing on metastasis rates and MVI positivity rate. The results demonstrated that the metastasis rate in *Cs* ^+^ HCC patients was significantly higher than in *Cs^-^* HCC patients (58.5% vs. 44.3%, *p* = 0.007) (**Figure 5A**). Likewise, the MVI positivity rate was also markedly higher in *Cs* ^+^ HCC patients compared to their *Cs* ^-^ counterparts (59.6% vs. 47.6%, *p* = 0.025) (**Figure 5B**). What’s more, we co-cultured *Cs-*derived excretory/secretory products (*Cs*ESPs) with MHCC-97H cells, and the control group was treated with PBS. It was found that the MHCC-97H cells treated with *Cs*ESPs had a greater migration ability than PBS (**Figure 5C**). The *Cs*ESPs co-culture group exhibited a significantly higher number of transmembrane cells, regardless of the presence of Matrigel, compared to the control group (**Figure 5D**). Finally, we further evaluated the effects of *Cs*ESPs on HUVECs tube formation. As expected, tube formation of HUVECs was significantly increased in the presence of *Cs*ESPs as demonstrated by the increase in total tube numbers and lengths (**Figure 5E**). Additionally, we investigated whether *Cs* affects tumor proliferation in HCC. The results showed no significant difference in the number of Ki67-positive cells between *Cs* ^+^ HCC patients and *Cs* ^−^ HCC patients (**Supplementary Figure S5A**). Furthermore, *Cs*ESPs had no significant proliferative effect on MHCC-97H cells (**Supplementary Figure S5B**). Collectively, our results suggest that *Cs* infection could promote HCC migration, invasion and angiogenesis.

## 4 Discussion

*Cs* infection is highly prevalent in southern China and has been classified as a class I carcinogen by the International Agency for Research on Cancer (8, 9, 30, 31). Although recent studies have linked *Cs* infection to HCC poor outcomes, the specific molecular mechanisms influencing HCC progression are still not largely clear (14, 32). The carcinogenic process of *Cs* involves multiple factors, including mechanical injury, abnormal immune responses, and inflammation triggered by the release of *Cs*ESPs (31, 33). In our study, by combining RNA sequencing and clinical data from HCC patients, along with *in vitro* cell experiments, we found that *Cs* infection could significantly increase HCC migration, invasion and angiogenesis. Our results provide a novel mechanism for *Cs* promoting HCC progression from a genetic perspective.

Previous studies indicate that during the progression of *Cs* infection, the host’s mRNA expression profile undergoes significant alterations, which are closely associated with impairments in host biological functions (18–20, 34). In this study, through mRNA-seq analysis of *Cs* ^+^ and *Cs* ^-^ HCC patients tumor samples, *Cs* infection induces different RNA expression profiles of tumors in HCC. Moreover, the DEGs between *Cs* ^+^ and *Cs* ^-^ HCC tumors were enriched in pathways related to ECM remodeling and immunosuppression, similar to the findings of Yapeng Qi et al. in their *Cs*-infected rat HCC model, where the DEGs identified in their study were also involved in ECM remodeling and liver fibrosis (20). Approximately 80%-90% of HCC occur in an environment of fibrosis and abnormal ECM, where tumor cell-derived ECM plays a critical role in cancer progression by modulating matrix stiffness, cell adhesion, vasculogenic mimicry, the immunosuppressive microenvironment, and cancer stemness (35–38). Interestingly, our *in vitro* cell experiments and clinical data also confirm that *Cs* infection promote tumor migration, invasion and angiogenesis in HCC. These factors may be important contributors to the poor prognosis observed in *Cs*-infected HCC patients.

In the late stage of infection, *Cs* could trigger the type 2 immune responses, which could accelerate tumor progression (11, 39, 40). The majority of HCC is the result of immune and chronic inflammation, characterized by a distinctive immunesuppressive microenvironment dominated by immune suppressor cells, such as dendritic cells, T regulatory cells (Tregs) and impaired T cells (41–43). Zheng-Jun Zhou and Li Pang et al. reported that a high density of tumor-infiltrating DCs is linked to increased infiltration Tregs and is associated with poor prognosis in HCC patients (44, 45). Young-Il Jeong et al. reported that *Cs*-derived total protein can promote an increase in the number of Tregs and DCs in a murine asthma model (46). Indeed, our data showed that resting DCs were significantly more abundant in *Cs^+^* HCC tumors. Moreover, the T-cell stimulant-related genes CD27 and ICOS were significantly down-regulated in *Cs^+^* HCC tumors. This suggests that the poor prognosis of *Cs*-infected HCC is linked to DCs accumulation and impaired T cell activity in the immune response.

In an effort to uncover the interactions among the DEGs, we constructed a PPI network and found that COL1A1 and COL1A2 were the top two significantly up-regulated interaction genes in *Cs^+^* HCC tumors, both of which encode type I collagen (47). Numerous studies have shown that COL1A1 plays a key role in angiogenesis and desmoplasia, with its overexpression linked to the invasion process in HCC (48, 49). And COL1A2 is associated with HCC stemness and unfavorable prognosis (50). In the study by Md Hafiz Uddin, Ji Hoon Jeong, and their team, it was similarly reported that there was a significant increase in type I collagen in *Cs*-infected intrahepatic cholangiocarcinoma of hamsters, as well as in *Cs*ESPs-treated H69 cells (51, 52). Moreover, TFs are key regulators that shape the interactions between cancer cells and their surrounding microenvironment, playing a crucial role in tumor behavior and progression (53). In this study, SOX17 and NFYC were the top two enriched TFs that could be identified as potential regulatory factors for *Cs*-infected HCC. SOX17 is a transcription factor involved in the early stages of cancer, orchestrating an immune-evasive program that facilitates cancer initiation and progression while also regulating tumor angiogenesis (54, 55). NFYC promotes Akt signaling in HCC by activating MTFR2, which has a significant impact on tumor growth, metastasis, and metabolic reprogramming (56).

Metabolic reprogramming, a key hallmark of cancer, is closely linked to the progression of HCC (57, 58). Su Han, Yang Yuan Qiu, and their team found that *Cs* can cause significant metabolic changes in the rat and rabbit model (59, 60). Lixia Xu et al. found that *Cs* could promote ICC progression through inducing metabolic alterations in malignant cells (31). In this study, we found that genes related to OXPHOS were up-regulated in *Cs^+^* HCC tumors compared to *Cs^-^* HCC tumors. OXPHOS is found to be up-regulated in many cancers, and targeting OXPHOS is proving to be an effective strategy for treating these cancer subtypes (61). Furthermore, studies have demonstrated that upregulation of OXPHOS is linked to increased migration and proliferation of liver cancer cells (62, 63). In our study, genes related to the electron transport chain and V-ATPase involved in OXPHOS were significantly up-regulated. Notably, the upregulation of genes ATP6V1E1 and ATP6V1H associated with V-ATPase subunits facilitates ECM degradation and cancer cell invasion by acidifying the extracellular environment and activating matrix metalloproteinases (64–66). These results indicate that the upregulation of OXPHOS may be a mechanism through which *Cs* influences the progression of HCC and could serve as a potential therapeutic target.

The prognosis of HCC is complicated by its intricate molecular characteristics and dynamic tumor microenvironment (41, 67). Our previous study has shown that *Cs* infection is significantly associated with poor prognosis of HCC (14). In this study, we found that *Cs* alters the mRNA profile in HCC, and the high-expression group of the top 10 up-regulated genes between *Cs^+^* HCC tumors and *Cs* ^-^ HCC tumors is associated with a significantly worse prognosis compared to the low-expression group in the LIHC-TCGA cohort. Therefore, we can conclude that the top 10 up-regulated genes between *Cs^+^* HCC tumors and *Cs* ^-^ HCC tumors are risk factors for poorer prognosis in *Cs*-infected HCC patients and may serve as potential biomarkers for the prognosis of *Cs*-infected HCC.

Collectively, our RNA-seq analysis results indicate that *Cs* may promote HCC migration, invasion, and angiogenesis by altering the tumor microenvironment and inducing metabolic reprogramming. Furthermore, our findings were validated through clinical retrospective analysis and *in vitro* experiments. These results demonstrate that *Cs*-infected HCC exhibits a distinct tumor microenvironment and molecular signatures, offering valuable insights into the carcinogenic potential of *Cs*. Nonetheless, these results should be interpreted with caution and strengthened by large multicenter randomized controlled studies. Additionally, the specific mechanisms by which it promotes HCC migration, invasion and angiogenesis in still need to be further investigated. In our future research, we can further investigate the key regulatory TFs, immune responses, and metabolism-related genes that *Cs* promotes in HCC, as well as genes linked to poor prognosis mentioned in this study. This will help identify potential therapeutic targets for the treatment of *Cs*-infected HCC.

## Data Availability

The datasets presented in this study can be found in online repositories. The names of the repository/repositories and accession number(s) can be found below: PRJNA1173109 (SRA).

## Supplementary material

Supplementary Table S1 Baseline Clinical Characteristics of Patients in the Retrospective Study.

Supplementary Table S2 Baseline Characteristics of Patients with Collected Clinical Tissue Samples.

Supplementary Table S3 The gene expression levels of tumors in Cs^+^ HCC and Cs^-^ HCC.

Supplementary Figure 1 Different immune microenvironment between *Cs* ^+^ HCC tumors and *Cs* ^-^ HCC tumors.

Supplementary Figure 2 Schema of metabolic pathways (glycolysis and electron transport chain [OXPHOS]) with differential gene expression of mRNA level between *Cs* ^+^ HCC tumors and *Cs* ^-^ HCC tumors.

Supplementary Figure 3 Differential gene expression of lncRNA level between *Cs* ^+^ HCC tumors and *Cs* ^-^ HCC tumors.

Supplementary Figure 4 Distinct RNA profiles between *Cs^+^* and *Cs^-^* HCC adjacent non-tumor tissues.

Supplementary Figure 5 The impact of *Cs* infection on HCC proliferation.
