# Supplementary material for: Clonorchis sinensis-infected hepatocellular carcinoma exhibits distinct tumor microenvironment and molecular features
Source: Front Immunol. 2025 Mar 17;16:1526699. doi: 10.3389/fimmu.2025.1526699 (PMC11955701; doi:10.3389/fimmu.2025.1526699)
Supplement: Supplementary Figure 1 — Different immune microenvironment between Cs + HCC tumors and Cs - HCC tumors. [file DataSheet1.doc]

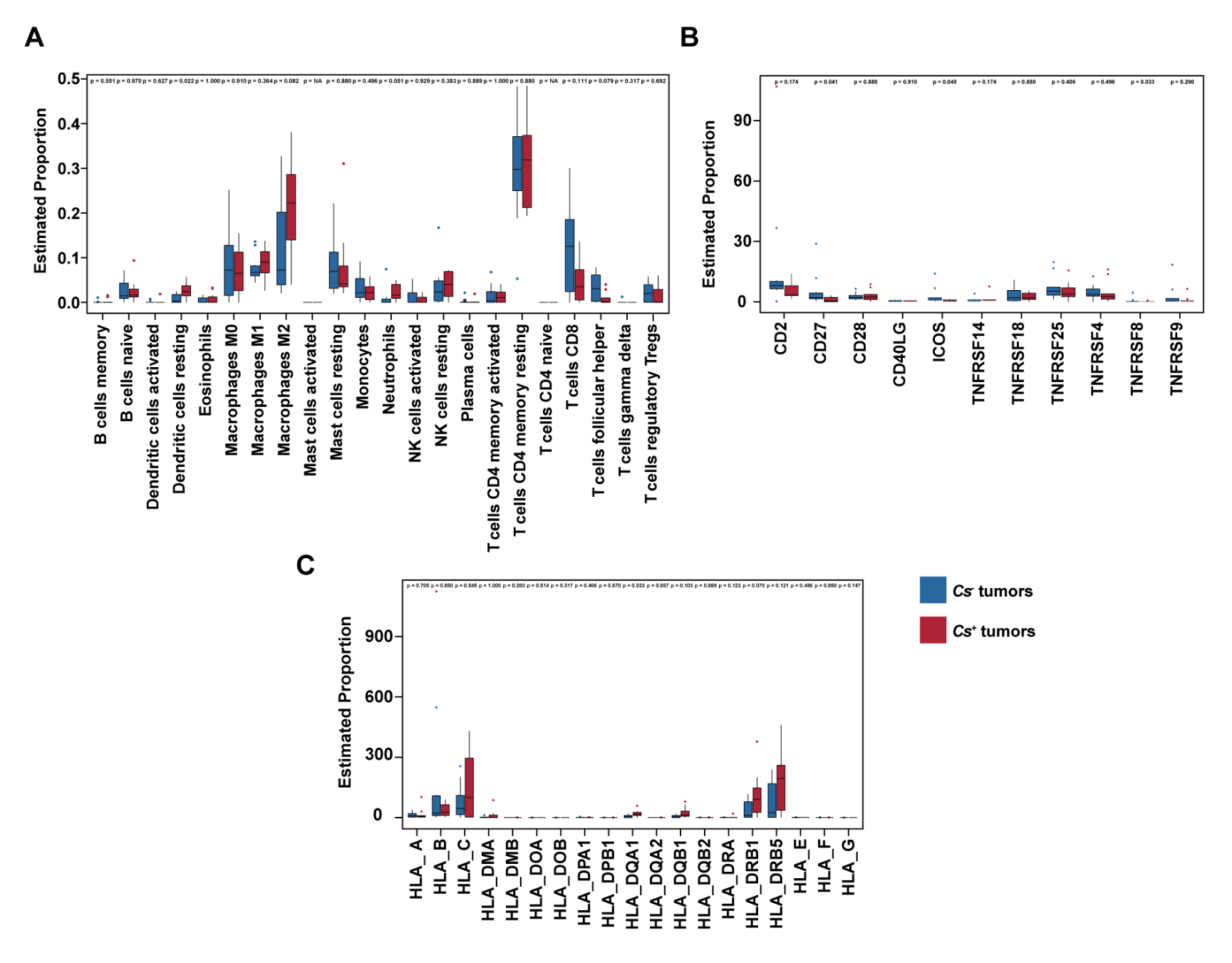


**Fig. S1. Different immune microenvironment between** ***Cs^+^* HCC tumors and *Cs^-^* HCC tumors.** **(A)** Different immune landscape between *Cs^+^* HCC tumors and *Cs^-^* HCC tumors. Different expression level of T-cell stimulant-related **(B)** and HLA-related **(C)** genes between *Cs^+^* HCC tumors and *Cs^-^* HCC tumors.


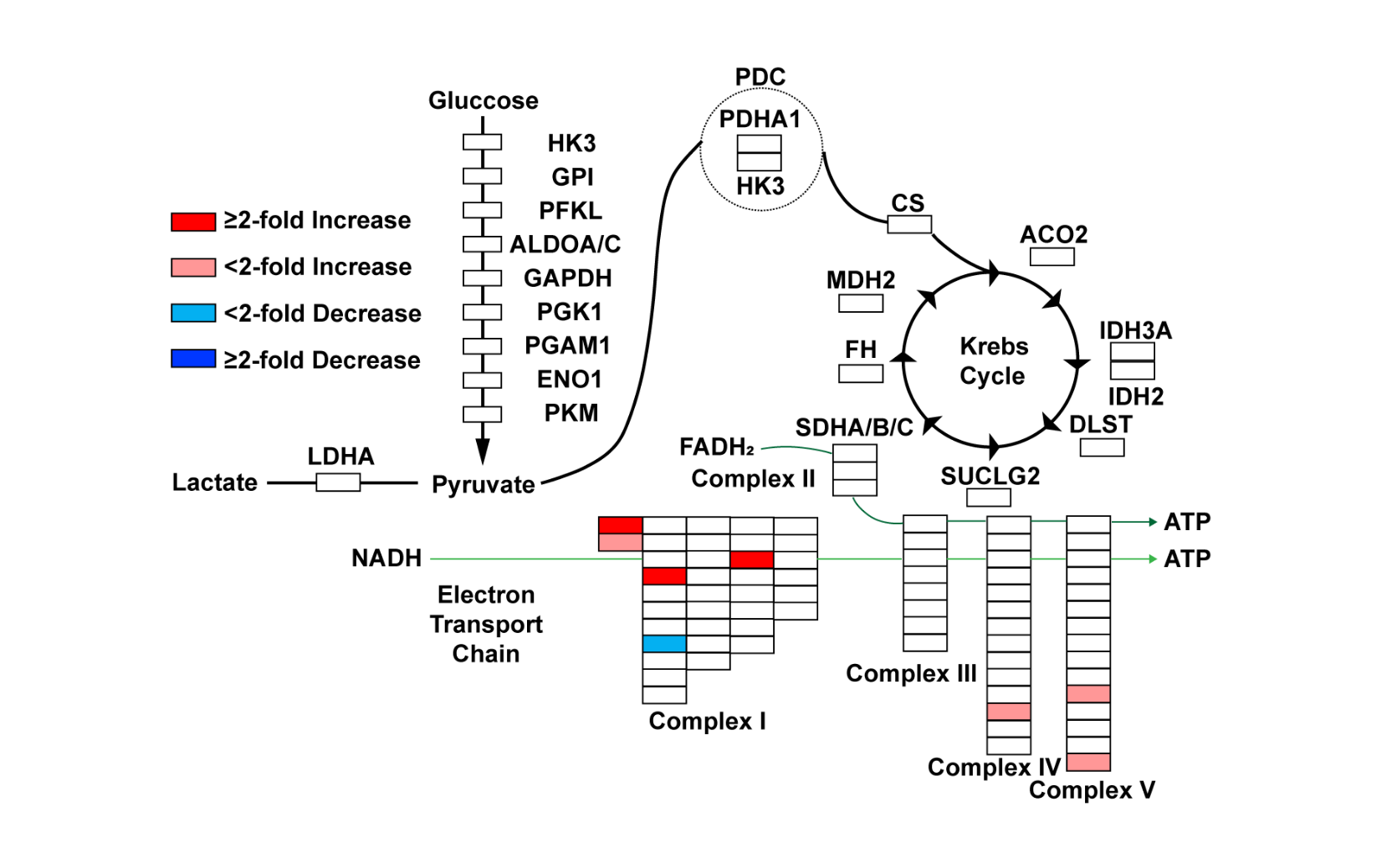


**Fig. S2. Schema of metabolic pathways (glycolysis and electron transport chain [OXPHOS]) with differential gene expression of mRNA level between *Cs^+^* HCC tumors and *Cs^-^* HCC tumors.**


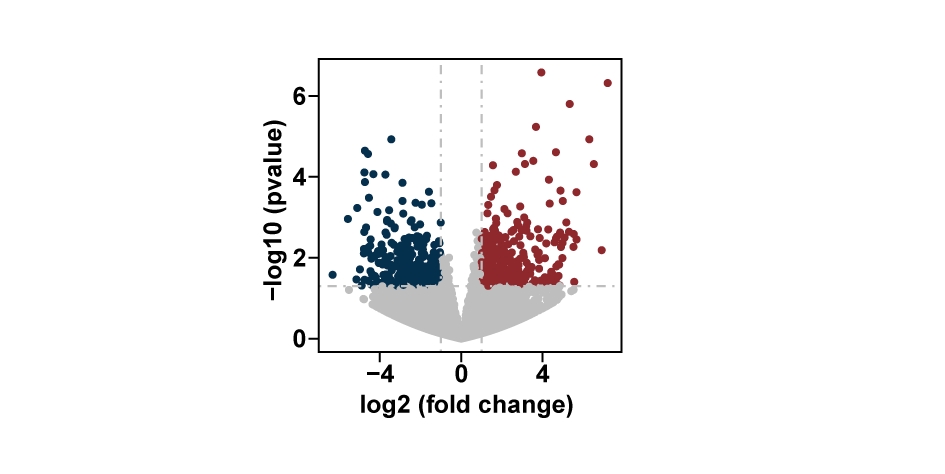


**Fig. S3. Differential gene expression of lncRNA level between *Cs^+^* HCC tumors and *Cs^-^* HCC tumors.**


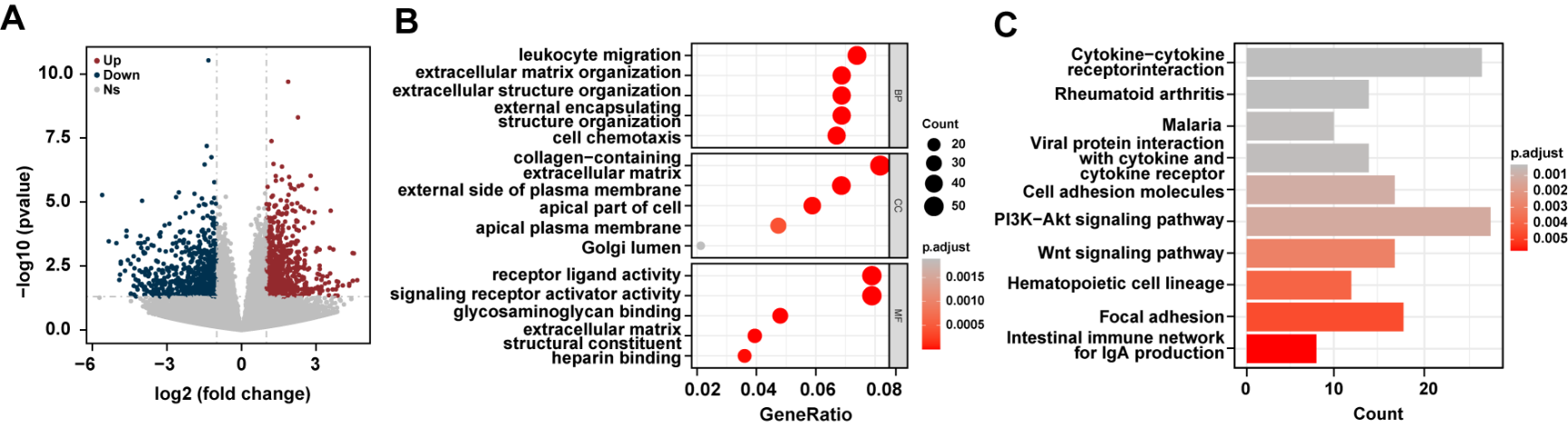


**Fig. S4. Distinct RNA profiles between *Cs^+^* and *Cs^-^* HCC adjacent non-tumor tissues. (A)** Volcano diagram of DEGs between *Cs^+^* and *Cs^-^* HCC adjacent non-tumor tissues. **(B)** Enrichment analysis of GO terms for up-regulated genes between *Cs^+^* and *Cs^-^* HCC adjacent non-tumor tissues. **(C)** KEGG analysis of up-regulated genes between between *Cs^+^* and *Cs^-^* HCC adjacent non-tumor tissues.


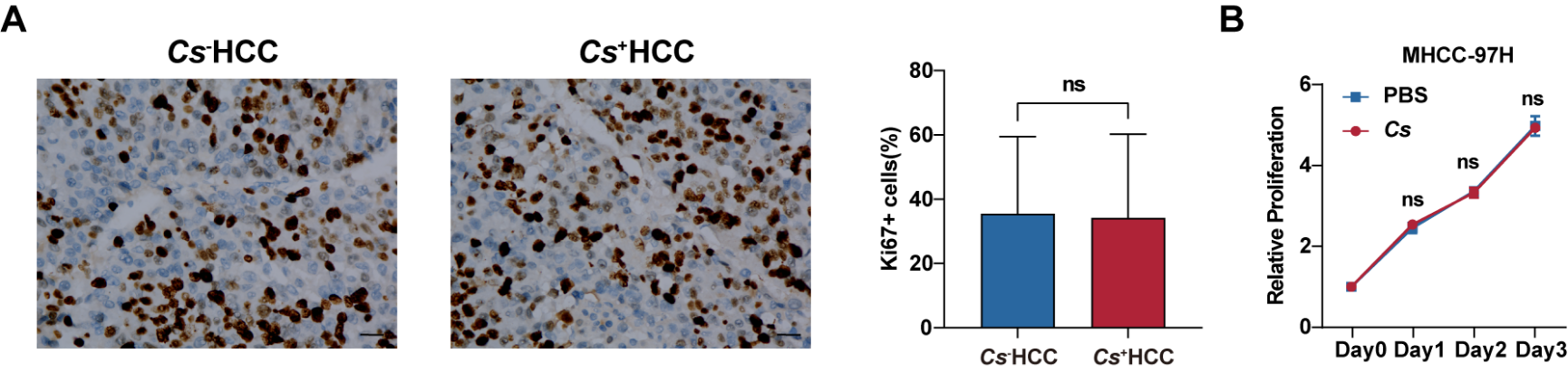


**Fig. S5. The impact of *Cs* infection on HCC proliferation. (A)** Comparison of the percentage of Ki67-positive cells between *Cs*^+^ HCC and *Cs^-^* HCC patients, along with representative pathological images (Scale bar: 50 μm) (*n*= 3). **(B)** The CCK8 assay of MHCC-97H cells co-cultured with *Cs*ESPs or PBS. **(A, B):** Data are presented as means ± SD;. ns: no significance
